# Assessment of Interrater and Intrarater Reliability of Cervical Range of Motion (CROM) Goniometer

**DOI:** 10.1155/2020/8908035

**Published:** 2020-06-12

**Authors:** Andzelina Wolan-Nieroda, Agnieszka Guzik, Paulina Mocur, Mariusz Drużbicki, Andrzej Maciejczak

**Affiliations:** ^1^Medical Faculty, The University of Rzeszów, Kopisto 2A Avenue, 35-959 Rzeszow, Poland; ^2^Department of Neurosurgery, St Luke Hospital, Lwowska 178 Street, 33-100 Tarnow, Poland

## Abstract

**Purpose:**

The current study was designed to assess interrater and intrarater validity of cervical range of motion measurements performed with a CROM goniometer. *Material and Methods.* The study involved 95 healthy university students (31 males and 64 females) aged 20-24 years. Two examiners performed measurements of cervical range of motion using a CROM goniometer. The same subjects were examined again after two weeks, in the same conditions. The results acquired by one rater during the first and the second examination were compared for reproducibility, while the results obtained by the two examiners were compared to assess validity and reliability of the tool. Cronbach's alpha was applied to determine intrarater reliability, and the values of correlations were used to assess the interrater agreement.

**Results:**

Analysis of the results showed both intrarater and interrater agreement in all the measures of cervical range of motion. The highest intrarater and interrater concordance was observed in the measure of extension. Intrarater agreement for Examiner 1 was reflected by Cronbach's alpha = 0.85, and for Examiner 2 by Cronbach's alpha = 0.89. As for the interrater agreement in the measure of extension, the value of correlation in both the first and the second measurement amounted to *r* = 0.89.

**Conclusions:**

Measurements performed with the CROM goniometer show interrater and intrarater agreement in assessments of cervical range of motion. The CROM goniometer can be recommended for use in daily clinical practice.

## 1. Introduction

The most common dysfunctions of the locomotor system include dysfunction of the cervical spine [1, 2], which is recognised as the most mobile segment of the spine [3]. Both the biomechanics and anatomy of the cervical spine contribute to the risk of strain, particularly affecting the intervertebral disc. This leads to various ailments generally referred to as neck pain syndrome [4]. Cervical spine disorders are common and they significantly impact quality of life, also constituting a problem for healthcare systems. Patients with cervical spine disorders usually experience pain, and reduced active and passive cervical ranges of motion. Recovery of physiological mobility is of key importance in cervical spine disorders [5].

The range of motion is a commonly accepted parameter used in assessment and evaluation of spine movements [6], yet it is also thought to be extremely difficult to access accurately due to the complex anatomy and the associated movements. The cervical range of motion is frequently applied as a baseline and outcome measure in documenting the effects of interventions, and in adjusting treatment plans in physiotherapy [7]. Physiotherapists and physicians can routinely perform assessment of active cervical range of motion in order to estimate the level of mobility limitation and its association with pain and to evaluate effects of treatment [8]. Many tools have been developed to enable measurement of cervical mobility, ranging from simple visual examination to the complex three-dimensional mobility assessment [5].

To be clinically useful, a measure of cervical range of motion must be confirmed for validity, reliability, and reproducibility [9]. Reproducibility reflects to what degree the results identified with a given device are stable over time, if the relevant subjects do not change. Reliability, the most frequently evaluated aspect, is a quality showing to what extent patients are differentiated, despite measurement errors [8]. Many other tools have been described in the literature as possible options for clinical evaluation when choosing an adequate method of assessing the cervical range of motion, yet there is no agreement as to which is the gold standard method for measurement of range of motion [10, 11].

Cervical range of motion (CROM) goniometer is a clinically available tool enabling straightforward measurement of neck mobility [7]. Review of literature shows there are a lot of studies focusing on CROM goniometer [5, 7–10, 12–22]. Williams et al., in a systematic review of reliability and validity studies focusing on methods designed to measure active and passive cervical range of motion, found that most of the relevant studies involved asymptomatic individuals examined by allied health professionals examining active range of motion. Devices that were reported to have “good” reliability and validity included the CROM device, the Spin-T goniometer, and a single inclinometer. Williams et al. established that the CROM device was highly reliable as regards all cervical spine movements (ICCs = 0.58-0.99) and presented good validity when compared to a gold standard (X-ray) (ICCs = 0.82-0.98). A conclusion drawn by these authors is that a significant number of reliability and concurrent validity studies have been published for CROM devices. Consequently, the CROM device has been most extensively evaluated and has been proven to provide a clinimetrically sound outcome measure [9]. Tousignant et al. conducted a criterion validity study of the CROM device focusing specifically on rotational range of motion in healthy adults. They compared range of motion measurements performed with the CROM device and an optoelectronic system. The values of cervical rotational range of motion acquired using the CROM showed a good to excellent linear relationship with the values obtained using the optoelectronic system: right rotation, *r* = 0.89, and left rotation, *r* = 0.94. Similar results were also identified in the case of the values reflecting flexion/extension and lateral flexion range of motion. In conclusion, the CROM presented excellent criterion validity for cervical rotation measurements [12]. In an earlier study, Tousignant et al. assessed the CROM goniometer for the criterion validity in a healthy population, focusing exclusively on the cervical flexion and extension. In this case, a Pearson *r* correlation test was applied to examine the criterion validity of the CROM goniometer in comparison to the radiographic method. The findings showed significant correlations between the two measurements (flexion: *r* = 0.97, *p* < 0.001; extension: *r* = 0.98, *p* < 0.001). In conclusion, the authors pointed out that the CROM goniometer provides valid measurements of cervical flexion and extension in healthy subjects [13]. Capuano-Pucci et al. reported satisfactory intratester and intertester reliability of the CROM and pointed out numerous advantages of the tool, such as ease of application and reliability. Their findings showed that the intrarater reliability (Pearson *r*), in the case of two raters using the CROM in a group of healthy subjects, ranged from 0.63 to 0.90 and from 0.62 to 0.91 for rater 1 and rater 2, respectively. Out of the 12 coefficients, three were below 0.80 [14]. Similarly, Hole et al. investigated the reliability and concurrent validity of two instruments intended for cervical range of motion measurement, i.e., CROM and single inclinometer, applied in healthy individuals. Their findings confirmed reliability of the CROM and single inclinometer. Concurrent validity of both these instruments was shown for flexion/extension and lateral flexion but not for rotation. Using both measuring devices, it was found that cervical mobility in all the planes of motion decreased with age but was not significantly related to gender [15]. Likewise, Nilsson assessed the intrarater reliability of the CROM used in healthy individuals. In this case however, the study focused on passive cervical range of motion, and the mean results from five consecutive measurements were taken into account as the raw data [16]. Furthermore, Love et al. assessed the interexaminer and the intraexaminer reliability in the measurement of passive flexion and extension ranges of motion of the cervical spine, carried out with an inclinometer and a CROM instrument. The examinations were performed by three chiropractic students. The intraexaminer reliability identified in the case of both the CROM and the inclinometer was high (ICC 95%CI = 0.96-0.99, ICC 95%CI = 0.85-0.96). The analyses focusing on interexaminer reliability also identified significant correlations (ICC 95%CI = 0.96-0.97, ICC 95%CI = 0.80-0.89) for the CROM and inclinometer, respectively. The authors concluded that although cervical passive range of motion can more effectively be measured with the CROM device, the findings also show satisfactory intraexaminer and interexaminer agreements for both instruments, confirming their clinical usefulness particularly for practitioners managing patients with injuries [17]. Finally, Audette et al. provided supporting evidence for the validity and test-retest reliability of CROM. Their study compared two measuring devices, i.e., CROM and the Fastrak motion analysis system, and showed that the values acquired with these yielded Pearson correlation coefficients in the range from 0.93 to 0.98. Their findings also showed good test-retest reliability of the measurements of cervical range of motion performed with the CROM device, which was reflected by the intraclass correlation coefficients (ICC) ranging from 0.89 to 0.98. The authors also showed that the measurements carried out with the CROM device were reliable in all movement directions [7].

Reliability and validity of the CROM device were also determined for patients with cervical spine dysfunction [18–22]. Law and Chiu examined the validity and reliability of CROM goniometer in measurements of cervical spine mobility in adult patients with and without neck pain. Their findings showed high intratester and intertester reliability in both the normal and chronic neck pain groups, with ICC values in the range of 0.75-0.92. The authors drew a conclusion that the cervical goniometer was reliable as a tool designed to measure cervical mobility in three planes in both neck pain and nonneck pain subjects. Construct validity of the CROM device was confirmed because the test results reflected significant differences in cranial range of motion between the subjects with neck pain and the controls [18]. Similarly, Fletcher and Bandy determined intrarater reliability of CROM measurement of cervical spine active range of motion in persons with and without neck pain. It was established that the CROM should be considered as a clinically reliable tool for measurement of cervical spine active range of motion [10]. Generally, the ICCs were the same across the two groups, and all the ICC values exceeded 0.80. These results show that the quality of the intrarater reliability for the measurement of cervical spine active range of motion using the CROM can be characterized as good to excellent [10]. The results reported by Fletcher and Bandy are similar to earlier findings related to intrarater reliability where the average ICCs were higher than 0.80 [19]. An investigation of interrater and intrarater reliabilities in cervical active range of motion assessment was also carried out by taking into account three measurement methods: a CROM instrument, a universal goniometer, and visual estimation. Youdas et al. reported that goniometric measurements of active cervical range of motion performed by the same physical therapist had ICCs exceeding 0.80 when the assessment was carried out with the CROM device or the universal goniometer on patients with cervical spine conditions [19]. Likewise, in a subsequent study focusing on normal cervical active range of motion, carried out with the use of the CROM, Youdas et al. [20] reported ICC values for intrarater reliability in healthy individuals. Most ICC values were 0.80 or higher, but the authors also reported ICCs which were significantly lower: 0.23 for flexion, 0.58 for right rotation, and 0.60 for right lateral flexion [20]. A study by Wibault et al. assessed the test-retest reliability of a CROM device in patients with cervical radiculopathy due to disc disease, as well as the criterion validity between the CROM device and a laser in neck-healthy subjects. The examinations were conducted by two physiotherapists and three students of physiotherapy trained in the testing procedure. The findings provide supporting evidence for effectiveness of the CROM device to be used in the clinical practice in assessing individuals with disc disease-related cervical radiculopathy. On the other hand, the criterion validity between the CROM device and a laser in neck-healthy subjects was questionable [21]. It has also been pointed out that there is a significant correlation between an Inertial Measurement Unit and a CROM device applied for the assessment of craniocervical movement in patients with cerebral palsy and healthy individuals [22].

Currently, in Poland there are no data showing validity of the CROM goniometer. This observation provided a motivation for the present study, and this article is the first research report related to this issue in our country.

The purpose of the study was to assess intrarater and interrater validity of cervical range of motion measurements performed with a CROM goniometer.

## 2. Material and Methods

### 2.1. Participants

Those eligible to participate in the study were healthy subjects, students of physiotherapy, with no cervical spine dysfunctions, which was confirmed by medical examination. Patients with pain symptoms, spinal disorders, and concurrent spinal mobility illnesses were excluded from the research.

### 2.2. Measurements

The cervical range of motion was examined using the CROM goniometer [7, 23, 24]. The examinations were performed in a Spine Kinesiology facility. The range of motion measurement was carried out with CROM goniometer by two independent examiners, during the same time and in the same conditions. To ensure the greatest reliability of the measurements, the examiners did not contact each other. The examiners are referred to as 1A and 2P.

Examination of mobility was repeated two weeks later in the same subjects and in the same conditions; the examination was carried out by the same examiners to enable evaluation of reproducibility of the measurements performed with the CROM goniometer (after two weeks: Examiner 3A and Examiner 4P).

**Figure 1:**
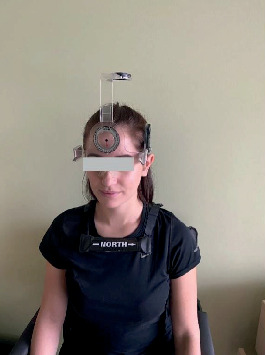
Measurement of the range of cervical spine motion using the CROM goniometer.

The measurements of the cervical range of motion, carried out with the CROM goniometer, were performed in all the planes of motion, with an accuracy of 1° (Figure 1). The goniometer was placed on the subject's head and a magnetic collar was attached to their shoulders; it was always arranged in the same position with respect to the magnetic pole. During all the measurements, the subjects were seated, with their back straight, and looking ahead; their feet were supported on the floor. The subjects remained in the same sitting position from the moment the recording procedure was started. The measurement was performed during “half a cycle” of the movement, i.e., from “the neutral” position to the final range of motion in a given plane. Each measurement of the active range of motion was preceded with instructions, whereby the examiner presented the movement to be performed by the subject. Subsequently, the subject performed a practice trial. During each trial, the movement was executed to reflect the subject's maximum capabilities, i.e., up to the moment they could feel pain or stretching of the tissues, or until substitution of movement occurred. When the maximum movement was performed in one direction, the final position was identified and recorded [7].

### 2.3. Statistical Analysis

Intrarater reliability was assessed by comparing two measurements carried out by one examiner. Assessment of intrarater reliability was performed using Cronbach's alpha coefficient whose value should not be lower than 0.6. Evaluation of interrater agreement involved comparison of measurements performed by two examiners, during the first session (1A versus 2P) and during the second session two weeks later (3A vs 4P). Assessment of interrater agreement was based on the value of correlation (*r*), which should not be lower than 0.5. Interrater and intrarater reliability of cervical range of motion (CROM) goniometer was assessed by calculating ICC (intraclass correlation coefficient). Level of agreement was interpreted as follows: ICC < 0.4—low level of agreement; ICC of 0.4-0.6—moderate level of agreement; ICC of 0.6-0.75—high level of agreement; and ICC of 0.75-1—very high level of agreement [25]. A two-tailed test for significance of structure indicators was applied to determine whether there were statistically significant differences by gender, age, and year of the course. Significance level was set to *p* < 0.05.

The sample selection calculator was used to calculate the minimum sample size, taking into account the number of people studying physiotherapy. A fraction size of 0.8 was applied, with a maximum error of 6%, and as a result a sample size of 92 people was determined.

## 3. Results

**Figure 2:**
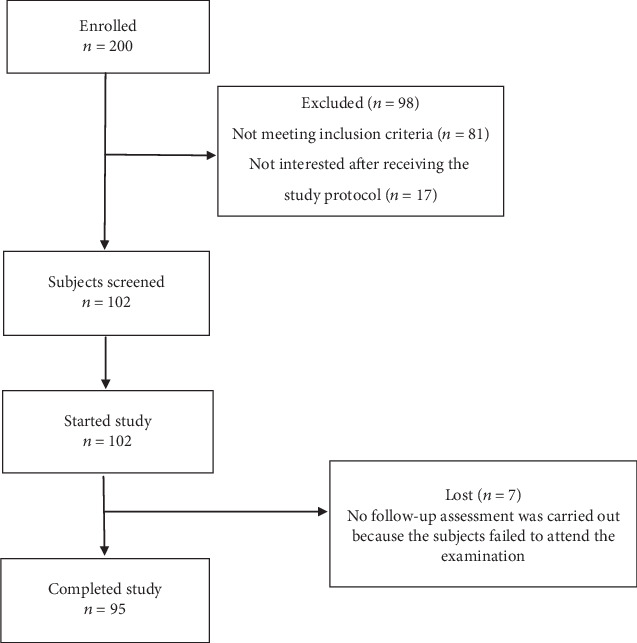
Flow of subjects through the study.

The flow of the subjects through the study is shown in Figure 2. No adverse events were observed during the study.

The study involved 95 healthy subjects. The subjects' mean age was 21 years, range of 20-24 years. The group included 31 males and 64 females. No significant differences were found in relation to age (*p* = 0.282) or year of the course (*p* = 0.282). There was a statistically significant difference related to gender; the study group comprised significantly more females than males (*p* = 0.023).

Based on the results of the above examinations, Table 1 presents the findings related to intrarater and interrater reliability in the measure of flexion. It was shown that intrarater reliability in the measure of flexion for Examiner A was reflected by Cronbach's alpha = 0.75 and in the case of Examiner P was expressed by Cronbach's alpha = 0.64 and for the ICC the values were 0.575 and 0.406, respectively. Interrater agreement in the measure of flexion during Exam I performed by Examiners 1A and 2P is shown by the values *r* = 0.72 and for the ICC = 0.701; in the case of Exam II, performed by Examiners 3A and 4P, it is indicated by the values *r* = 0.5 and for the ICC = 0.460.

Analysis of the results related to intrarater and interrater reliability in the measure of extension showed that intrarater reliability in the measure of extension for Examiner A was reflected by Cronbach's alpha = 0.85, and for the ICC value = 0.644, and that of Examiner P was expressed by Cronbach's alpha = 0.89 and for the ICC value = 0.796. Interrater agreement in the measure of extension during Exam I performed by Examiners 1A and 2P and in Exam II performed by Examiners 3A and 4P is shown by the values *r* = 0.89 and for the ICC = 0.808 and ICC = 0.878, respectively. Results related to intrarater and interrater reliability in the measure of extension are presented in Table 2.

Table 3 presents the results related to intrarater and interrater reliability in the measure of left lateral flexion. It was shown that intrarater reliability in the measure of left lateral flexion for Examiner A was reflected by Cronbach's alpha = 0.7, for the ICC value = 0.528, and in the case of Examiner P, it was expressed by Cronbach's alpha = 0.75, for the ICC value = 0.520. Interrater agreement in the measure of left lateral flexion during Exam I performed by Examiners 1A and 2P is shown by the values *r* = 0.61, for the ICC = 0.552, and in the case of Exam II, performed by Examiners 3A and 4P, it is indicated by the values *r* = 0.71, for the ICC = 0.696.

Analysis of the results related to intrarater reliability and interrater agreement in the measure of right lateral flexion showed that intrarater reliability in the measure of right lateral flexion for Examiner A was reflected by Cronbach's alpha = 0.72, for the ICC value = 0.550 and in the case of Examiner P was expressed by Cronbach's alpha = 0.64, for the ICC value = 0.405. Interrater agreement in the measure of right lateral flexion during Exam I performed by Examiners 1A and 2P is shown by the values *r* = 0.82, for the ICC = 0.742, and in the case of Exam II, performed by Examiners 3A and 4P, is indicated by the values *r* = 0.72, for the ICC = 0.712 (Table 4).

Analysis of the results related to intrarater reliability and interrater agreement in the measure of left rotation showed that intrarater reliability in the measure of left rotation for Examiner A was reflected by Cronbach's alpha = 0.65, for the ICC value = 0.462, and in the case of Examiner P was expressed by Cronbach's alpha = 0.66, for the ICC value = 0.472. Interrater agreement in the measure of left rotation during Exam I performed by Examiners 1A and 2P is shown by the values *r* = 0.67, for the ICC = 0.48, and in the case of Exam II, performed by Examiners 3A and 4P, is indicated by the values *r* = 0.74, for the ICC = 0.712 (Table 5).

Comparison of the results related to intrarater and interrater reliability in the measure of right rotation showed that intrarater reliability in the measure of right rotation for Examiner A was reflected by Cronbach's alpha = 0.67, for the ICC value = 0.510, and in the case of Examiner P was expressed by Cronbach's alpha = 0.72, for the ICC value = 0.535. Interrater agreement in the measure of right rotation during Exam I performed by Examiners 1A and 2P is shown by the values *r* = 0.66, for the ICC = 0.533, and in the case of Exam II, performed by Examiners 3A and 4P, is indicated by the values *r* = 0.72, for the ICC = 0.702 (Table 6).

## 4. Discussion

The study was carried out to assess reliability and reproducibility of CROM goniometer measurements. The assessments were performed in three planes, in subjects showing no cervical spine dysfunctions. The current findings show good interrater and intrarater reliability of the CROM goniometer. The ICC in all the measures exceeded the required value, reflecting good intrarater and interrater reliability. Cronbach's alpha coefficient reflected good intrarater reliability of the CROM goniometer. Likewise, the value of correlations in all the measurements exceeded the minimum necessary to confirm interrater agreement of CROM goniometer measurements.

There are numerous reports in the literature discussing reliability of CROM [5, 7, 8, 10]. In Poland, however, there are no publications related to this subject matter. This observation provided a motivation to carry out the present study on Polish ground.

Chantal et al. assessed reliability, validity, and responsiveness of devices applied in measurement of cervical mobility, including the CROM goniometer. Their research shows that, given its well-established clinimetric properties, the device may easily be used in daily clinical practice [8]. According to Audette et al., given the emphasis on the importance of objective tools, the CROM goniometer may be recommended for use in assessing cervical mobility both for the needs of research and in rehabilitation contexts. The reported findings confirmed good reliability of the CROM goniometer; the authors also assessed error of measurements and reported good test-retest reliability of the device [7]. Fletcher and Bandy published a study focusing on intrarater reliability of the tool in subjects with and without neck pain. Their findings are consistent with those reported in the present study. They documented sufficient reproducibility of CROM goniometer measurements, demonstrating that the device can effectively be used in daily clinical practice in patients with neck pain and in subjects with no symptoms of cervical problems [10]. Mangone et al. presented comparable results related to reliability and reproducibility of cervical range of motion measurements performed with CROM goniometer. The study involved healthy individuals, with no spinal dysfunctions. These authors emphasize the importance of such factors as the affordability of a CROM device and the simplicity of its operation, as well as its portability owing to the small size of the tool [5]. Rondoni et al. published a review which was designed to assess intrarater and interrater reliability of active cervical range of motion measurements performed with expensive technological equipment as compared to affordable common use devices. The study involved subjects with nonspecific neck pain. The authors additionally investigated whether the reliability of the measuring device was influenced by the plane of the assessed movement. Conclusions from this review suggest that the reliability of the assessment is not significantly affected by the type of the measuring device; hence, a comparison of more expensive and more affordable devices showed no significant effects. Likewise, the plane of movement does not influence the results in cervical range of motion measurements [24].

The current findings show satisfactory results in the movements for all the planes, i.e., sagittal, frontal, and transverse, while the best results were observed in the assessment of extension, in terms of both interrater and intrarater agreement. Likewise, highly significant statistical correspondence was found in the measurement of left lateral flexion. The lowest values could be observed in measurements of left and right rotation; however, even in this case, the results were higher than the minimum values required for interrater and intrarater reliability.

Similarly, Bush et al. demonstrated that the most consistent CROM measurements are found in assessments of flexion, extension, and lateral flexion performed with an inclinometer. The measurements were carried out by 34 practicing physiotherapists who examined three persons. The measures of the range of flexion, extension, and lateral flexion were compared with results shown in X-ray pictures, while the ranges of rotation were compared with axial tomography scans [26]. In another study, Dhimitri et al. also obtained good results for flexion and extension, in assessment of interrater and intrarater reliability of upper cervical motion measurements performed with a CROM goniometer in a group of 30 healthy individuals aged from 23 to 37 years [27]. Likewise, Capuano-Pucci et al. in a study involving 20 healthy subjects and assessing interrater and intrarater reliability of CROM goniometer observed the best results in the measures of flexion and extension [14].

While investigating other methods of cervical range of motion measurement, researchers point out that clinical usefulness of such complex devices as electrogoniometers and digital inclinometers is doubtful due to practical reasons and costs involved. Dynamic X-ray examination of intervertebral motion for years was recognised as the “gold standard” in assessment of cervical range of motion; today, however, applicability of this invasive method is limited due to the small number of X-ray images which can be obtained while ensuring minimum exposure of the patient to radiation. Another method, i.e., the three-dimensional kinematic analysis, is also known for the accuracy of the calculations; however, it is also expensive and it can be used only in laboratory settings and require experienced personnel [3, 5, 28].

The study limitation is to narrow down the researched patients to one age group. Follow-up research encompassing various age groups, especially 50 plus one, seems to be necessary to be conducted.

In view of the above, it appears from the analysis of the acquired findings that assessment of cervical motion with CROM goniometer results in highly reliable and reproducible measurements which are easy to perform and does not require significant financial resources. Notably, this is the first scientific report related to this subject matter in Poland, and it provides clinicians with an additional tool which can be applied in the daily practice. In order to validate the findings, further studies should be conducted in groups of subjects with various cervical spine dysfunctions.

## 5. Conclusion

Measurements performed with CROM goniometer show interrater and intrarater agreement in assessments of cervical range of motion.

The CROM goniometer can be recognised as an alternative tool which can be recommended for use in daily clinical practice.

## Figures and Tables

**Table 1:** Intrarater and interrater agreement in the measure of flexion.

Measure		Mean	SD	Cronbach's alpha (intrarater reliability)	Pearson's product-moment correlation coefficient	ICC (interrater and intrarater reliability)	95% CI		Agreement
Flexion	1A 3A	55.70 58.27	8.05 8.18	0.75	0.60	0.575	0.341	0.743	Moderate
	2P 4P	53.97 61.60	9.72 8.07	0.64	0.48	0.406	0.128	0.632	Moderate
	1A 2P	55.70 53.97	8.05 9.72	—	0.72	0.701	0.514	0.825	High
	3A 4P	58.27 61.60	8.18 8.07	—	0.50	0.460	0.195	0.663	Moderate

**Table 2:** Intrarater and interrater agreement in the measure of extension.

Measure		Mean	SD	Cronbach's alpha (intrarater reliability)	Pearson's product-moment correlation coefficient	ICC (interrater and intrarater reliability)	95% CI		Agreement
Extension	1A 3A	84.17 90.87	11.14 15.17	0.85	0.77	0.644	0.303	0.813	High
	2P 4P	88.33 89.63	15.75 13.36	0.89	0.81	0.796	0.656	0.883	Very high
	1A 2P	84.17 88.33	11.14 15.75	—	0.89	0.808	0.628	0.898	Very high
	3A 4P	90.87 89.63	15.17 13.36	—	0.89	0.878	0.787	0.931	Very high

**Table 3:** Intrarater and interrater agreement in the measure of left lateral flexion.

Measure		Mean	SD	Cronbach's alpha (intrarater reliability)	Pearson's product-moment correlation coefficient	ICC (interrater and intrarater reliability)	95% CI		Agreement
Left lateral flexion	1A 3A	50.20 52.10	7.00 9.20	0.70	0.55	0.528	0.281	0.711	Moderate
	2P 4P	47.70 52.47	9.75 7.06	0.75	0.63	0.520	0.209	0.721	Moderate
	1A 2P	50.20 47.70	7.00 9.75	—	0.61	0.552	0.311	0.727	Moderate
	3A 4P	52.10 52.47	9.20 7.06	—	0.71	0.696	0.506	0.822	High

**Table 4:** Intrarater and interrater agreement in the measure of right lateral flexion.

Measure		Mean	SD	Cronbach's alpha (intrarater reliability)	Pearson's product-moment correlation coefficient	ICC (interrater and intrarater reliability)	95% CI		Agreement
Right lateral flexion	1A 3A	47.03 48.87	5.92 7.12	0.72	0.57	0.550	0.309	0.725	Moderate
	2P 4P	45.27 50.00	8.91 6.91	0.64	0.48	0.405	0.122	0.626	Moderate
	1A 2P	47.03 45.27	5.92 8.91	—	0.82	0.742	0.570	0.851	High
	3A 4P	48.87 50.00	7.12 6.91	—	0.72	0.712	0.530	0.832	High

**Table 5:** Intrarater and interrater agreement in the measure of left rotation.

Measure		Mean	SD	Cronbach's alpha (intrarater reliability)	Pearson's product-moment correlation coefficient	ICC (interrater and intrarater reliability)	95% CI		Agreement
Left rotation	1A 3A	73.00 66.00	14.65 14.01	0.65	0.57	0.462	0.193	0.665	Moderate
	2P 4P	61.73 69.57	13.91 13.60	0.66	0.56	0.472	0.198	0.680	Moderate
	1A 2P	73.00 61.73	14.65 13.91	—	0.67	0.48	0.420	0.811	Moderate
	3A 4P	66.00 69.57	14.01 13.60	—	0.74	0.712	0.529	0.832	High

**Table 6:** Intrarater and interrater agreement in the measure of right rotation.

Measure		Mean	SD	Cronbach's alpha (intrarater reliability)	Pearson's product-moment correlation coefficient	ICC (interrater and intrarater reliability)	95% CI		Agreement
Right rotation	1A 3A	74.00 77.83	9.92 10.14	0.67	0.60	0.510	0.200	0.695	Moderate
	2P 4P	72.67 77.50	9.80 9.29	0.72	0.67	0.535	0.289	0.699	Moderate
	1A 2P	74.00 72.67	9.92 9.80	—	0.66	0.533	0.298	0.701	Moderate
	3A 4P	77.83 77.50	10.14 9.29	—	0.72	0.702	0.518	0.822	High

## Data Availability

The datasets used to support the findings of this study have been deposited in the Library of Rzeszow University repository (https://repozytorium.ur.edu.pl/handle/item/5215).

## Abbreviations

CROM: cervical range of motion.

## References

[1] Wolański W., Tejszerska D. (2009). Biomechanical analysis of the cervical spine in a situation of stabilization. *Engineering Modeling*.

[2] Chaves T. C., Nagamine H. M., Belli J. F. C., de Hannai M. C. T., Bevilaqua-Grossi D., de Oliveira A. S. (2008). Confiabilidade da fleximetria e goniometria na avaliação da amplitude de movimento cervical em crianças. *Brazilian Journal of Physical Therapy*.

[3] Porzych P., Ratuszek-Sadowska D., Pyskir M., Simińska J., Ogórkowski K., Kitschke E. (2016). Cervical vertebral column - mobility and selected ways of measurement - a literature review. *Journal of Education*.

[4] Mikołajczyk E., Jankowicz-Szymańska A., Guzy G., Maicki (2013). The impact of comprehensive physiotherapy on the functional state of patients with pain in the cervical spine. *Hygeia Public Health*.

[5] Mangone M., Bernetti A., Germanotta M. (2018). Reliability of the cervical spine device for the assessment of cervical spine range of motion in asymptomatic participants. *Journal of Manipulative and Physiological Therapeutics*.

[6] Jonas R., Demmelmaier R., Hacker S. P., Wilke H. J. (2018). Comparison of three-dimensional helical axes of the cervical spine between in vitro and in vivo testing. *Spine Journal*.

[7] Audette I., Dumas J. P., Côté J. N., de Serres S. J. (2010). Validity and between-day reliability of the cervical range of motion (crom) device. *The Journal of Orthopaedic and Sports Physical Therapy*.

[8] de Koning C. H. P., van den Heuvel S. P., Staal J. B., Smits-Engelsman B. C. M., Hendriks E. J. M. (2008). Clinimetric evaluation of active range of motion measures in patients with non-specific neck pain: a systematic review. *European Spine Journal*.

[9] Williams M. A., McCarthy C. J., Chorti A., Cooke M. W., Gates S. (2010). A systematic review of reliability and validity studies of methods for measuring active and passive cervical range of motion. *Journal of Manipulative and Physiological Therapeutics*.

[10] Fletcher J. P., Bandy W. D. (2008). Intrarater reliability of CROM measurement of cervical spine active range of motion in persons with and without neck pain. *The Journal of Orthopaedic and Sports Physical Therapy*.

[11] Lachtman D. S., Bartha D. A., Beltran M. M. (2015). Rater reliability and concurrent validity of single and dual bubble inclinometry to assess cervical lateral flexion. *Journal of Manipulative and Physiological Therapeutics*.

[12] Tousignant M., Smeesters C., Breton A. M., Breton E., Corriveau H. (2006). Criterion validity study of the cervical range of motion (CROM) device for rotational range of motion on healthy adults. *The Journal of Orthopaedic and Sports Physical Therapy*.

[13] Tousignant M., de Bellefeuille L., O'Donoughue S., Grahovac S. (2000). Criterion validity of the cervical range of motion (CROM) goniometer for cervical flexion and extension. *Spine*.

[14] Capuano-Pucci D., Rheault W., Aukai J., Bracke M., Day R., Pastrick M. (1991). Intratester and intertester reliability of the cervical range of motion device. *Archives of Physical Medicine and Rehabilitation*.

[15] Hole D. E., Cook J. M., Bolton J. E. (1995). Reliability and concurrent validity of two instruments for measuring cervical range of motion: effects of age and gender. *Manual Therapy*.

[16] Nilsson N. (1995). Measuring passive cervical motion: a study of reliability. *Journal of Manipulative and Physiological Therapeutics*.

[17] Love S., Gringmuth R. H., Kazemi M., Cornacchia P., Schmolke M. (1998). Interexaminer and intraexaminer reliability of cervical passive range of motion using the CROM and Cybex 320 EDI. *The Journal of the Canadian Chiropractic Association*.

[18] Law E. Y., Chiu T. T. (2013). Measurement of cervical range of motion (CROM) by electronic CROM goniometer: a test of reliability and validity. *Journal of Back and Musculoskeletal Rehabilitation*.

[19] Youdas J. W., Carey J. R., Garrett T. R. (1991). Reliability of measurements of cervical spine range of motion—comparison of three methods. *Physical Therapy*.

[20] Youdas J. W., Garrett T. R., Suman V. J., Bogard C. L., Hallman H. O., Carey J. R. (1992). Normal range of motion of the cervical spine: an initial goniometric study. *Physical Therapy*.

[21] Wibault J., Vaillant J., Vuillerme N., Dedering A., Peolsson A. (2013). Using the cervical range of motion (CROM) device to assess head repositioning accuracy in individuals with cervical radiculopathy in comparison to neck- healthy individuals. *Manual Therapy*.

[22] Carmona-Pérez C., Garrido-Castro J. L., Torres Vidal F. (2020). Concurrent validity and reliability of an inertial measurement unit for the assessment of craniocervical range of motion in subjects with cerebral palsy. *Diagnostics*.

[23] Davies M., Moore I. S., Moran P., Mathema P., Ranson C. A. (2016). Cervical range of motion, cervical and shoulder strength in senior versus age-grade Rugby Union International front-row forwards. *Physical Therapy in Sport*.

[24] Rondoni A., Rossettini G., Ristori D. (2017). Intrarater and inter-rater reliability of active cervical range of motion in patients with nonspecific neck pain measured with technological and common use devices: a systematic review with meta-regression. *Journal of Manipulative and Physiological Therapeutics*.

[25] Cicchetti D. V. (1994). Guidelines, criteria, and rules of thumb for evaluating normed and standardized assessment instruments in psychology. *Psychological Assessment*.

[26] Bush K. W., Collins N., Portman L., Tillett N. (2013). Validity and Intertester reliability of cervical range of motion using inclinometer measurements. *The Journal of Manual & Manipulative Therapy*.

[27] Dhimitri K., Brodeur S., Croteau M., Richard S., Seymour C. J. (1993). Reliability of the cervical range of motion device in measuring upper cervical motion. *Journal of Manual & Manipulative Therapy*.

[28] Carvalho G. F., Chaves T. C., Gonçalves M. C. (2014). Comparison between neck pain disability and cervical range of motion in patients with episodic and chronic migraine: a cross-sectional study. *Journal of Manipulative and Physiological Therapeutics*.

